# A Novel Computer-Assisted Method of Bite Mark Analysis for Gender Determination

**DOI:** 10.1155/2018/7130876

**Published:** 2018-10-09

**Authors:** Anirban Maji, Tanya Khaitan, Rupam Sinha, Soumyabrata Sarkar, Pratik Verma, Anjani Kumar Shukla

**Affiliations:** ^1^Department of Oral Medicine and Radiology, Haldia Institute of Dental Sciences and Research, Haldia, West Bengal 721645, India; ^2^Department of Oral Medicine and Radiology, Dental Institute, Rajendra Institute of Medical Sciences, Ranchi, Jharkhand 834009, India; ^3^Department of Orthodontics & Dentofacial Orthopaedics, Dental Institute, Rajendra Institute of Medical Sciences, Ranchi, Jharkhand 834009, India

## Abstract

**Background:**

Bite mark analysis is an imperative area of forensic odontology and considered the commonest form of dental evidence presented in the criminal court. The process of comparing bite marks with a suspect's dentition includes analysis and measurement of shape, size, and position of an individual's teeth. The present study was aimed to evaluate the bite marks of males and females using a novel indirect computer-assisted method and explicate its application in forensic odontology.

**Materials and methods:**

60 subjects (30 males and 30 females) with normal occlusion were included in the present study. Bite registrations were obtained with help of modelling waxes, and positive replicas were prepared with dental stone and barium powder. Intraoral periapical radiographs were taken for the same. The radiographs obtained were scanned and analyzed by measuring tools using Sidexis Next Generation software. Intercanine distance (ICD), line AB, angle ABX, and angle ABY were measured. The Kruskal–Wallis test was performed to compare the bite marks of males and females.

**Results:**

The mean ICD of males and females was found to be 32.95 mm and 29.84 mm, respectively, and was statistically highly significant with a *p* value <0.001. The mean ICD, line AB, and angle ABX were found to be higher in males when compared to females.

**Conclusion:**

Analysis of bite marks using this novel computer-assisted method is a simple, reliable, easily reproducible, and economical technique with confidentiality of the identity of the participants involved.

## 1. Introduction

Odontologic evidence is the third most precise method of identification of fingerprints and DNA analysis in forensic sciences. “The criminal may lie through his teeth though teeth themselves cannot lie” is rightly said by Furness. Anything diverse, such as discrepancy from normality, becomes an important tool when trying to establish an identity of a suspect [1, 2].

Bite mark is “a mark made by the teeth either alone or in combination with other mouth parts” [3]. Mac Donald defined bite mark as “a representative pattern left in an object or tissue by the dental structures, either alone or in combination with other oral structures of an animal or human” [4]. According to the Old Testament, Adam was convinced by Eve to place “bite marks” in apple. The first bite mark case in Colonial America occurred during the Salem witch trials in 1692, where Rev. George Burroughs was convicted and hanged for witchcraft, including biting his victims. The contemporary history of bite marks is contemplated to have started with Sorup [3].

Bite mark analysis is based on the principle that “no two mouths are alike”. According to Pretty and Turnbull, the central doctrine of bite mark analysis is based on two assumptions; firstly that human teeth are unique and secondly that sufficient detail of the uniqueness is rendered during the biting process to facilitate identification [5, 6]. Distinctiveness and uniqueness of human dentition allows the forensic odontologists to achieve a strong judgment in cases of personal identification and bite mark analysis. Bite marks may unveil individual tooth marks, may present as a double-arched pattern or even a multiple overlying bruise. Bite marks can be deformed due to flexibility and elasticity of the skin. The amount of pressure given during bite, body position, and the angle of the maxilla and mandible during bite can alter the manifestation of a bite mark [7, 8].

Human bite marks are found when teeth are used as weapons of anger, excitement, control, or destruction. The impression can be left on the skin, chewing gum, pencils, or pens and also found on musical instruments, cigarettes, food materials such as cheese, fruit, potato, chocolate, and so on. These are encountered in cases of crimes especially homicides, quarrels, abduction, child abuse, and sexual assaults and during sports events and sometimes intentionally inflicted to frame someone fallaciously [8, 9]. The terms commonly used in bite mark analysis are victim—the recipient of the bite mark and perpetrator—the person who caused the bite mark [4]. While bite marks on the body are intentionally caused, those found on food articles are usually imperceptibly left by the offenders at the scene of the crime. So, as to recognize the offender, the dental casts of suspected persons are prepared using dental material and matched. Bite marks if analyzed properly can prove the involvement of a particular person or persons in a meticulous crime [9]. West et al. felt that bite marks on the human skin can be experimentally created to a level that permits comparison to bites delivered in combative or life-threatening situations, and more research is needed using living subjects to explore a variety of experimental situations [5]. One of the most remarkable, difficult, and sometimes troublesome challenges in forensic dentistry is the identification, recovery, and analysis of the bite marks with the suspected biters [9].

Various methods of bite mark analysis are present such as impression making from bitten substances by dental stone and hand tracing from dental study casts, photography method, photocopying, and computer-assisted overlay generation method [10]. Previous studies suggest that computer-generated overlays provided the most accurate and reproducible exemplars [11]. One of the parameters of the investigation is the measurements of the intercanine distance (ICD), as the impressions of the anterior teeth are usually the most evident and likely to be measurable [9]. With the above background, the present study was aimed to analyze and compare the bite marks of males and females via a novel indirect computer-assisted method using ICD and elucidate its application in forensic odontology.

## 2. Materials and Methods

The present study was carried out in the Department of Oral Medicine and Radiology at Haldia Institute of Dental Sciences and Research, Haldia, India, after the protocol had been approved by the institutional ethical committee. 60 subjects (30 males and 30 females) of age group 20–40 years of the same race and ethnicity on voluntarily basis (convenient sampling) were selected for the present study. Subjects with normal occlusion and presence of both maxillary canines were included. Patients with occlusal disharmony, previous/current orthodontic treatment, restorative procedures in maxillary canines, developmental anamoly of canines, history of trauma or lesion associated, and extreme age groups were considered in the exclusion criteria. The importance and need of the study was explained to all the subjects and informed consent obtained for the same.

Firstly, the subjects were asked to gently bite on a sheet of modelling wax and bite registrations recorded. The positive replicas of the bite marks were then prepared with the help of barium powder (to increase the radiodensity) and dental stone. Intraoral periapical radiographs were taken for the dental casts obtained. Further, the radiographs were scanned and the images analyzed using the computed-assisted method.

**Figure 1:**
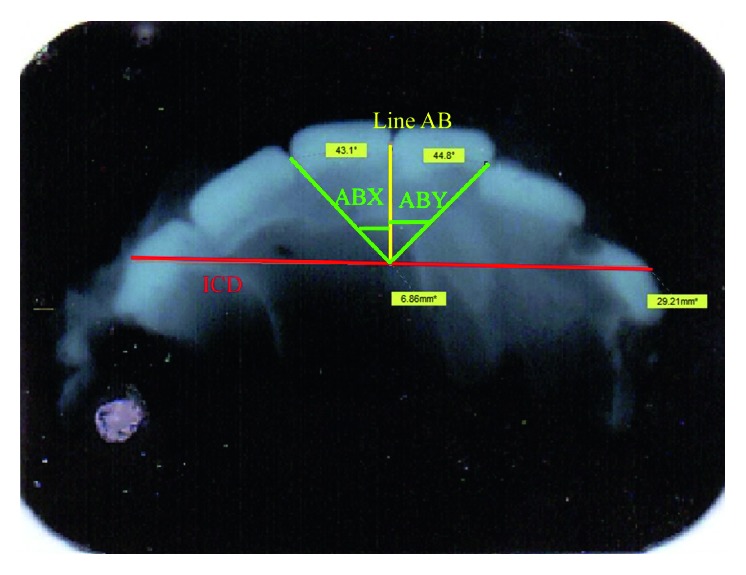
Measurement of ICD, line AB, and angles ABY and ABX radiographically using Sidexis Next Generation software.

With the help of Sidexis Next Generation software, the following parameters were measured. Intercanine distance (ICD) is a perpendicular line drawn from the midpoint of the upper central incisors to the intercanine line (line AB), and two lines (X and Y) drawn from both the distal aspect of the central incisors to the midpoint of line AB forming angles ABX and angle ABY, respectively, were measured (Figure 1). All the measurements obtained were recorded in a proforma specially designed for the study. The results obtained were subjected to statistical analysis using SPSS Version 16.01 (statistical package for social sciences) software. The Kruskal–Wallis test was used to compare the bite marks of males and females. *p* value <0.01 was considered significant.

## 3. Results

60 subjects (30 males and 30 females) with a mean age of 28.92 years were selected for the study. The mean ICD of males and females was found to be 32.96 mm and 29.84 mm, respectively, with a mean difference of 3.11 mm and statistically highly significant *p* value <0.001. The mean value of line AB in males was 9.30 mm and females was 8.63 mm with a mean difference of 0.67 which was statistically insignificant with a *p* value of 0.09. The mean differences of angle ABY and angle ABX in males and females were 0.25 and 0.24, respectively, which was also statistically insignificant (*p* value: 0.81 and 0.83, respectively). The mean ICD, line AB, and angle ABX were found to be higher in males when compared to females (Table 1).

## 4. Discussion

Bite marks are inimitable due to miscellany of the craniofacial parameters. It has been established as an efficient tool in identifying criminals involved particularly in abduction, child abuse, homicide, and sexual assaults. This analysis is by far the most demanding and complicated part of forensic odontology. Bite mark assessment should be carried out as earlier as possible, as the key features fades with time [10, 11].

Typically, a human bite mark comprises two opposing U-shaped arches separated by open spaces. A hematoma may occupy the centre space of the bite mark, caused by soft tissue compression during biting action [8]. These marks are usually caused by the incisors, canine, and premolars. If the executor has dentures, supplementary marks can be present, depending on the use of removable or fixed prosthesis [12]. Canine marks in a bite are the most prominent, and the normal distance between maxillary canines is 25–40 mm [13]. In the present study, incisors and canines were chosen as the guide for establishing a correlation with human bite marks, and canine marks were found to be more obvious. ICD proved to be an important parameter in the present study along with line AB and angle ABX for determining bite marks.

The appearance of the bite mark is prejudiced by multiple factors such as site and nature of the bite mark, amount of force/pressure used, and finally whether the victim was living or dead at the time of crime [13, 14]. The shape of the dental arch, intercanine distance, bucco-lingual version and mesial or distal drifting of teeth, spacing between teeth, rotation of teeth, curves of biting edges, missing teeth, developmental defects, restorations, and wear patterns also serve as various predisposing factors. These characteristics contribute to the uniqueness of each tooth making it different from the other [15, 16]. All these factors such as malalignment, developmental defects, and restored teeth were considered in the exclusion criteria in the present study.

Factors affecting the accuracy of bite mark analysis are the amount of biting pressure, proper impression technique, generation of dental casts, and quality and angulations of radiographs taken. Uniqueness is replicated on the bitten substrate in sufficient detail to enable a match to the individual under suspicion [8, 17]. Daniel et al. analyzed the bite marks using cheese, apple, and chocolate and found that the accuracy was greater for chocolate and cheese [1]. Patil et al. and Kashyap et al. recorded and determined bite marks using modelling wax which was also used in the present study [9, 13]. Sansare and Karjodkar assessed bite marks on various common foodstuffs (chocolate, apple, chewing gum, and cheese) for different time intervals using cone beam computed tomography (CBCT) to evaluate the dimensional changes in the foodstuffs. The highest accuracy was observed in chocolate followed by cheese, chewing gum, and apple. This CBCT-assisted analysis of bite marks was proved to be a nondestructive, accurate, and efficient method [18].

Literature reveals various methods for bite mark analysis such as manual, radiographic, and computed-assisted by different authors. Van der Velden et al., Patil et al., Rathode et al., Osman et al., and Gopal and Anusha used indirect methods for bite mark analysis and proved that computed-assisted methods were preferably better [6–8,12,13]. Kashyap et al. in 2015 also used indirect digital imaging and compared the bite mark pattern and intercanine distance between humans and dogs [9]. The present study introduced a novel computer-assisted method for bite mark analysis wherein the bite marks were registered using modelling wax sheetsand radiographs, taken of the positive replica, and further various parameters were assessed such as ICD, line AB, and angles ABY and ABX using Sidexis Next Generation software.

Reinprecht et al. suggested any maxillary intercanine distance >24.1 mm and <43.0 mm represented a human bite mark. This made a meaningful scientific contribution to the presentation of bite mark evidence in high cases of crime in South Africa [19]. The mean ICD was found to be 32.96 mm in males and 29.84 mm in females. Difference in mean ICDs among males and females was found to be highly significant. The variability of the ICD measurements found in both males and females had similar values, but on average, measurements for males were greater. Although mean values of line AB and angles ABY and ABX were insignificant, mean values of line AB and angle ABX were higher in males. In contrary, Tarvadi et al. evaluated that ICD as a parameter is an unreliable method for bite mark analysis [20].

Although a novel computer-assisted method was introduced for bite mark analysis in the present study, further studies should be carried out with a larger sample size in an attempt to clarify the origin and differentiation of biting injuries. Skilled expertise also has an influence on results but still this method has a high level of reliability.

## 5. Conclusion

Assessment of bite mark evidence made on humans requires additional investigation with development of sophisticated software with superior specificity as a tool to aid in justice. Advanced methods like DNA analysis give more precise results but is least followed due to its nonpracticability and cost efficiency. Comparatively, the present novel indirect computer-assisted method for bite mark analysis is simple, reliable, easily reproducible, economical, and less time consuming with confidentiality of identity and no risk to the participants. It is strongly recommended to discontinue hand-tracing methods which depend on subjective input by forensic odontologists and use computer-assisted methods which have comparatively higher reliability and accuracy. Bite mark analysis alone should not be allowed to lead to a guilty verdict although it offers the opportunity to exclude a suspect from a crime when the data do not correspond.

## Figures and Tables

**Table 1:** Mean values and mean differences of ICD, line AB, and angles ABY and ABX in males and females.

Parameters	Mean of males (M) and females (F)	Mean difference	Standard deviation	*t*-value	*p*-value
Intercanine distance (mm)	M-32.96 F-29.84	3.11	0.58	5.38	<0.001

Line AB (mm)	M-9.30 F-8.63	0.67	0.39	1.70	0.09

Angle ABY	M-47.49 F-47.25	0.25	1.03	0.24	0.81

Angle ABX	M-46.02 F-45.87	0.24	1.13	0.21	0.83
